# Improved Aerial Surface Floating Object Detection and Classification Recognition Algorithm Based on YOLOv8n

**DOI:** 10.3390/s25061938

**Published:** 2025-03-20

**Authors:** Lili Song, Haixin Deng, Jianfeng Han, Xiongwei Gao

**Affiliations:** 1 School of Information Engineering, Inner Mongolia University of Technology, Jinchuan Campus, Hohhot 010080, China; songlili@imut.edu.cn (L.S.); dhaixin2022@163.com (H.D.); gxw374940468@163.com (X.G.); 2 Inner Mongolia Key Laboratory of Intelligent Perception and System Engineering, Hohhot 010080, China

**Keywords:** aerial photograph, small object detection, floating object recognition, environmental monitoring

## Abstract

The water surface environment is highly complex, and floating objects in aerial images often occupy a minimal proportion, leading to significantly reduced feature representation. These challenges pose substantial difficulties for current research on the detection and classification of water surface floating objects. To address the aforementioned challenges, we proposed an improved YOLOv8-HSH algorithm based on YOLOv8n. The proposed algorithm introduces several key enhancements: (1) an enhanced HorBlock module to facilitate multi-gradient and multi-scale superposition, thereby intensifying critical floating object characteristics; (2) an optimized CBAM attention mechanism to mitigate background noise interference and substantially elevate detection accuracy; (3) the incorporation of a minor target recognition layer to augment the model’s capacity to discern floating objects of differing dimensions across various environments; and (4) the implementation of the WIoU loss function to enhance the model’s convergence rate and regression accuracy. Experimental results indicate that the proposed strategy yields a significant enhancement, with mAP50 and mAP50-95 increasing by 11.7% and 12.4%, respectively, while the miss rate decreases by 11%. The F1 score has increased by 11%, and the average accuracy for each category of floating objects has enhanced by a minimum of 5.6%. These improvements not only significantly enhanced the model’s detection accuracy and robustness in complex scenarios but also provided new solutions for research in aerial image processing and related environmental monitoring fields.

## 1. Introduction

Floating waste on the water surface can negatively impact environmental beautification, jeopardize the safety of water conservation and hydropower projects, and jeopardize the life and health of aquatic animals. As high-definition monitoring, unmanned vessels, and unmanned aerial vehicles (UAVs) mature, scholars have started to implement these technologies in water inspection. Fefilatyev, S. [1], Chen, J. [2], Socek, D. [3], Ma, Z. [4], Lin, Y. [5], Jin X. [6], and others have conducted valuable investigations into the detection of floating objects on the water surface using traditional image processing techniques based on data acquisition methods such as fixed high-definition monitoring and unmanned vessels. However, their algorithms are time-consuming and exhibit low universality. Zhang, L. et al. [7] introduced a Faster R-CNN technique that amalgamates low-level and high-level features, significantly enhancing the model’s detection accuracy for multi-scale floating objects. Lin, F. et al. [8] augmented the network’s feature extraction capability by integrating feature map attention at the conclusion of the YOLOv5 backbone network, thereby endowing the model with greater robustness against micro-occlusions and reflected water surface artifacts. Qiao, G. et al. [9] proposed the incorporation of coordinate attention into the backbone network and enhance the feature pyramid network [10] to increase the model’s performance in feature extraction. Yang, X. et al. [11] developed a way to incorporate the CA mechanism into the C3 module, significantly enhancing the model’s detection accuracy for floating objects. Li, H. et al. [12] suggested substituting the SE module in the MobileNet architecture with the CBAM module, which significantly diminished the impact of intricate backgrounds on detection accuracy. Li, K. et al. [13] introduced a strategy utilizing a scale-penalized intersection-union ratio to enhance the convergence speed and regression accuracy of the model. Tan, W. et al. [14] proposed a way to include the dark2 module into the backbone network and enhance its branch output structure, thereby improving the accuracy of small target identification. Yue, X. [15] and Xiang, X. [16] et al. proposed an approach that incorporates a target identification layer into YOLOv5s, thereby enhancing the accuracy and precision of floating object recognition on water surfaces. Shi, C. [17] recommended the incorporation of an upgraded C2f-CA module and a small target detection layer into YOLOv8n to improve the identification capability of small floating debris.

In comparison to the aforementioned detection methods for floating objects on the water’s surface, UAVs offer a broader range of vision, are unaffected by topographical constraints, and supply coordinate data for the subsequent removal of floating debris. Nonetheless, the floating objects in aerial pictures of water surfaces are diminutive and more challenging to identify. Guo, Y. [18] suggested a quick transformation technique that approximates MBD based on the investigation of detection algorithms from the viewpoint of UAV aerial photography, and the detection accuracy is up to 88%. This enhancement is confined to low-light water surface photographs against a plain background. Kong, H. [19] introduced a river duckweed extraction technique utilizing a semantic segmentation Res-UNet network, which enhanced detection accuracy; nevertheless, the anchor labeling process was time-consuming. Maharjan, N. et al. [20] introduced the YOLOv5s algorithm utilizing fine-tuning transfer learning [21], which offers an optimal balance between accuracy and computing complexity; nonetheless, its detection efficacy is contingent upon the visual properties of floating objects on the surface. Li, D. [22] developed a solution utilizing the FocalLoss function [23] as a substitute for the binary cross-entropy function. The enhanced algorithm maintains a commendable detection accuracy for aerial photos at altitudes beyond 20 m, with a mean Average Precision (mAP) value of 67.36%. Du, X. et al. [24] proposed a method for integrating SimAM attention into the skip connection feature pyramid network SCFPN, which significantly improves the feature representation capability of floating objects on the water surface. Chiang, C. et al. [25] introduced a technique involving the incorporation of 22 supplementary filters to the neck of the YOLOv5 model, resulting in an enhanced confidence rate of 98.86%. This experiment was limited to an aerial height of 5 m and was unable to effectively identify floating objects on the ocean surface in aerial photos captured at greater altitudes. Huang, J. et al. [26] proposed the incorporation of a micro-scale detection layer and enhancements to the CBAM during the feature fusion phase to facilitate the identification of floating objects on water surfaces at elevated altitudes in aerial photography. This significantly improved the model’s capacity to identify floating objects in rivers, attaining a mean Average Precision (mAP) value of 91.8%. Deng, Y. et al. [27] advocated the introduction of the FocalNext module, Context Aggregation attention mechanism, and Decouple Head to address the challenges given by the irregular, diversified, and varying sizes of floating debris. The mean Average Precision (mAP) of the enhanced model is 86.7%, representing a 4.1% increase over the benchmark model, thus significantly augmenting the model’s detection capability for small objects.

Although the aforementioned research significantly improves the model’s capacity to extract and express the characteristics of floating objects on the water surface, there are few studies that consider the perspective of UAVs. During the river patrol operation, it is essential to circumvent shoreline impediments of varying elevations, such as medium and small bridges [28], while contending with an often intricate water surface environment. To address the challenges posed by the altitude restrictions of UAVs—such as the small proportion of aerial surface floating objects in images, the weakened saliency of their features, and the reduced detection accuracy caused by lighting variations and mutual occlusion among floating objects. This paper aims to improve the detection accuracy of floating objects as its primary objective. Leveraging the efficiency and precision of YOLOv8n, we propose an enhanced YOLOv8-HSH algorithm for the detection and classification of floating objects in aerial images. This work aims to inspire further research in UAV-assisted environmental monitoring and to advance knowledge in model-assisted detection techniques for complex aquatic environments. The primary contributions are as follows:

1. Aiming at the problem that YOLOv8n has difficulty extracting the features of very small floating objects in complex environments, this paper proposes an improved HorBlock module. This improvement not only effectively enhanced the ability to detect dense small targets in aerial imagery but also has significant application value in fields such as environmental monitoring, maritime rescue, and floating debris quantity estimation.
2. Attaining accurate detection in noisy surroundings is essential in domains such as remote sensing, ecological monitoring, and search and rescue operations. This research examines the problems of excessive background noise in high-altitude aerial photographs and the inadequate detection accuracy of models for floating objects across various water color conditions. This paper proposes an enhanced similarity spatial attention module (SSAM). This technology exhibits extensive application potential in monitoring different water environments, providing a novel approach for target detection challenges in intricate settings.
3. To address the issue of insufficient detection performance of the YOLOv8n model for floating objects of varying sizes in complex environments, this paper redesigns the Head component by incorporating a small target detection layer, significantly enhancing the model’s detection accuracy and classification capabilities. This enhanced technique efficiently addresses the detection difficulties arising from substantial size changes of targets in aerial photos, offering a superior method for target detection in intricate situations.
4. This paper effectively improved the model’s ability to locate and classify floating objects by combining the dynamic adjustment mechanism of the WIoU loss function. This enhancement also improves the convergence speed and regression accuracy of the model.

## 2. Materials and Methods

### 2.1. Dataset Construction

**Figure 1:**
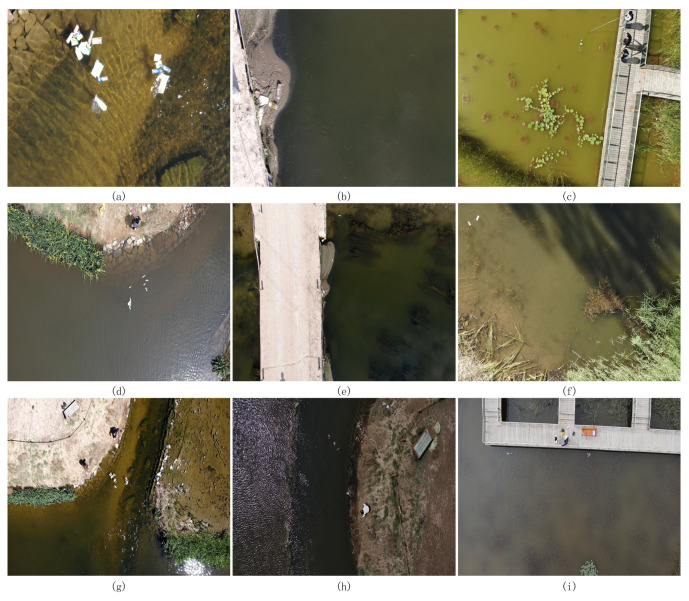
Example images of water surface floating objects captured at different heights, weather conditions, and seasons. From top to bottom, the aerial photography heights are 10 m, 15 m, and 20 m. Among them, (**a**,**b**) were captured using a 10 m zoom lens. (**b,e**), respectively, show images of the same location captured at different altitudes. (**c**,**i**), respectively, show images of the same water body captured during summer and autumn. (**d**,**g**), respectively, depict images of the same water body under different weather conditions. (**f**) demonstrates the different colored water bodies used in this study, (**h**), shows an anomalous image captured by the drone during an inspection on a cloudy day, where the river water appears black due to unknown reasons.

There is not yet a publicly available dataset of surface floating objects derived from UAV aerial photography. Consequently, this paper constructs a dataset that emulates the actual environment. The dataset utilizes the DJI M300RTK quadcopter outfitted with the DJI Zen Yuntai camera (H20, P1). Photographs of the rivers, lakes, and artificial lakes in Hohhot City and its neighboring villages were taken at flight heights of 10 m, 15 m, and 20 m. The dataset successfully collected 2200 aerial photos featuring floating objects on the water’s surface, encompassing variations in weather and lighting during spring, summer, and autumn. Some aerial images are shown in Figure 1. In the same water body, different seasons and weather conditions can lead to variations in water color. For instance, Figure 1c and Figure 1i, respectively, show images of the same water body captured during summer and autumn. In summer, the water appears green with a significant presence of aquatic plants, while in autumn, the water takes on a grayish-brown hue with almost no interference from aquatic plants. Similarly, Figure 1d and Figure 1g, respectively, depict images of the same water body under different weather conditions. The former was taken after rain, showing relatively turbid water, while the latter was captured on a sunny day, displaying clear water. The resolution of the captured images is 1920 × 1080. All photographs in the dataset do not employ any illumination enhancement technologies; the variations in illumination and blurring effects are naturally occurring phenomena resulting from the UAV’s mobile shooting procedure. Data annotation is completed by the LabelImg tool, and the training set and test set are divided according to the ratio of 8:2.

**Figure 2:**
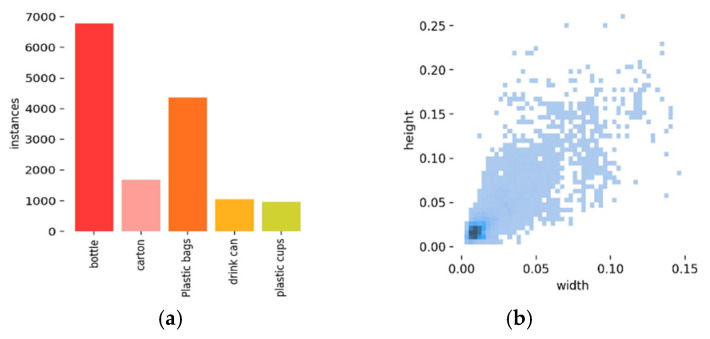
Label number and label distribution size diagram. (**a**) Tag quantity map; (**b**) label distribution size map.

The classification of floating objects on the water surface comprises five categories: bottles, boxes (including beverage, dairy packaging, and cigarette boxes), plastic bags, cans, and plastic cups (including disposable cups and bubble barrels). The quantity of labels and the distribution size of the labels are illustrated in Figure 2. As shown in Figure 2a, the dataset exhibits an imbalance in sample distribution. Specifically, the labels for plastic bottles and plastic bags are more abundant, while the labels for carton, drink cans, and plastic cups are less frequent and decrease sequentially. However, this label distribution aligns with the primary composition of floating waste on the water surface in real-world scenarios, reflecting the actual characteristics of floating waste [29]. Therefore, the imbalance in this dataset has a negligible impact on the model’s performance and does not significantly affect its overall effectiveness. Figure 2b illustrates that the label aspect ratio of the dataset in this work is significantly below 0.1, aligning with the notion of a small target.

### 2.2. Experimental Environment and Parameters

Experimental environment: The operating system used in the text is Windows 10; the CPU is an i7-12700K (Intel, United States), and the memory is 32 GB; the GPU is an RTX 3090 Ti (ASUS, China), and the memory is 24 GB. The deep learning framework is Pytorch 1.12.0 and CUDA 12.0.

The experimental parameters are established as follows: the input image dimensions are 1024 × 1024, the processing batch size is 8, the number of working threads is 8, the seed is 3407, and the training iterations total 300. The other parameters are the official default settings of YOLOv8. Each model used in all ablation and comparison experiments follows the above parameter configuration unless otherwise stated. This is carried out to make sure that model performance is fair and can be compared. This research conducts all experiments without the use of pre-trained weights.

### 2.3. General Framework

**Figure 3:**
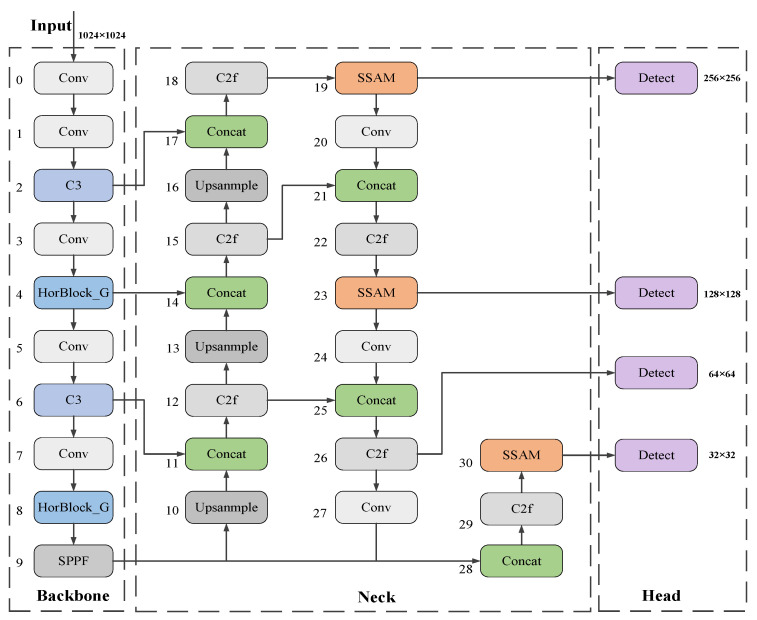
The network structure diagram of YOLOv8-HSH algorithm.

Figure 3 illustrates the enhanced algorithmic framework derived from the YOLOv8n network. In the backbone section, the C2f modules in the fourth and eighth layers are substituted with Horblock_G modules, which significantly improve the model’s capacity to extract features of minuscule floating objects on the water surface in intricate situations. Concurrently, C3 modules replace the C2f modules in the second and sixth levels, enabling multi-path information flow. This not only alleviates the computational burden of the backbone network but also enables the implementation of more complex network architectures, thereby enhancing the model’s performance and precision. Secondly, in the Neck component, this paper implements a lightweight SSAM attention mechanism to diminish the weights of less prominent features and amplify the model’s focus on salient features. Ultimately, this paper incorporates a 256 × 256 small target detection layer in the Head section, which markedly enhanced the detection and classification efficacy of floating objects of varying sizes at diverse aerial altitudes.

### 2.4. Horblock_G Module

**Figure 4:**
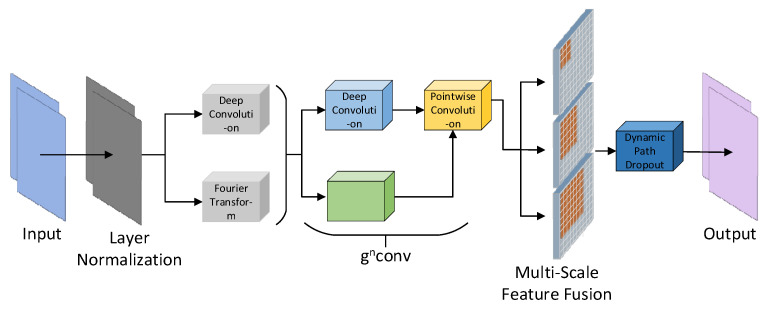
HorBlock_G network structure diagram.

**Figure 5:**
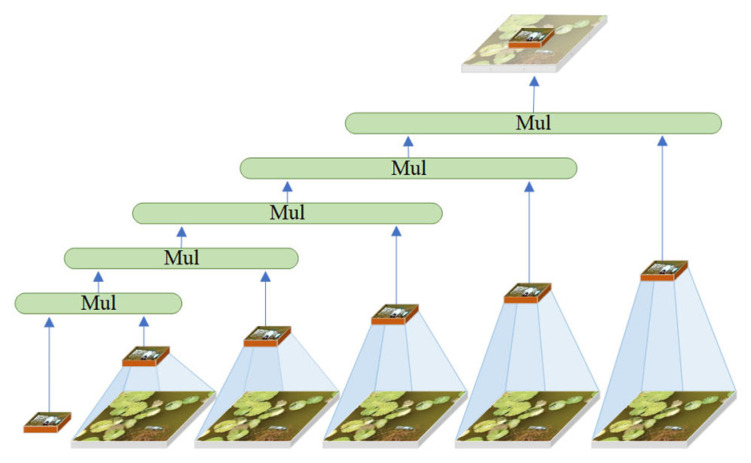
$g^{n}Conv$ implementation thought diagram.

Figure 4 illustrates the improved structure of the HorBlock [30,31] module. The fundamental operation of HorBlock_G is still based on recursive gated convolution $g^{n}Conv$. By repeatedly processing the identical water surface feature map, it selectively permits the passage of attention features, subsequently superimposing and enhancing these features with each iteration. This enables the model to discern more intricate feature relationships, thereby enhancing the feature extraction capability of floating objects on the water surface. The implementation idea is shown in Figure 5. However, the resolution of $g^{n}Conv$ in various spaces is inadequate due to its single-scale feature, and the issue of inadequate detection of small targets in intricate water surface images is constrained by aerial height. In this regard, HorBlock_G incorporates multi-scale feature fusion convolution modules with convolution kernels of 3 × 3, 5 × 5, and 7 × 7 on the basis of $g^{n}Conv$. This enables the extraction of more fine-grained floating object features on different spatial scales, the enhancement of the ability to capture extremely small targets in large feature maps, and the acquisition of detail features with greater context information retention. Furthermore, to mitigate the overfitting issue associated with complex models during training, HorBlock_G enhanced the original model’s fixed path discarding probability mechanism by implementing a dynamic path discarding strategy. This allows the model to adaptively modify the path discarding probability based on the current number of training iterations, facilitating broader exploration of the parameter space in the initial phase while emphasizing stability and convergence in the subsequent phase.

The operation process of high-order interaction and path discarding strategy of $g^{n}Conv$ is as follows:

(1) Calculate the first-order spatial interactive gated convolution gConv of $g^{n}Conv$.

Let $x\in R^{HW\times C}$ be the input feature, then the output $y=g^{n}Conv(x)$ is expressed as follows:

(1) $$[P_{0}^{HW\times C},q_{0}^{HW\times C}]=\phi_{in}(x)\in R^{HW\times 2C}$$

(2) $$p_{1}=ƒ(q_{0})⊙p_{0}\in R^{HW\times C}$$

(3) $$y=\phi_{\mathrm{out}}(p_{1})\in R^{HW\times C}$$

In the formula, $\phi_{in}$ and $\phi_{\mathrm{out}}$ are the linear projection layers mixed by the execution channels, ƒ is the deep convolution, $p_{0}$ is the projection feature input of the region of interest, $q_{0}$ is the projection feature input of the adjacent region, and $p_{1}$ is the projection feature after the first-order spatial interaction.

(2) High-order interaction is introduced to perform recursive gated convolution. The operation is as follows:

(4) $$[p_{0}^{HW\times C_{0}},q_{0}^{HW\times C_{0}},\ldots ,q_{n-1}^{HW\times C_{n-1}}]=\phi_{in}(x)\in R^{HW\times (C_{0}+\sum_{0\leq k\leq n-1}C_{k})}$$

(5) $$p_{k+1}={ƒ}_{K}(q_{k})⊙g_{k}(p_{k})/\alpha ,k=0,1,\cdot \cdot \cdot ,n-1$$

Among them, 1/α is used as a scaling factor to stabilize the training, α is set to 1, and k denotes the order. $\left\{g_{k}\right\}$ is used to match the dimensions of different orders, and $C_{k}$ is the channel dimension setting of each order, as follows:

(6) $$g_{k}=\left\{\begin{array}{ll} \mathrm{Identity}, & k=0\\ \mathrm{Linear}(C_{k-1},C_{k}), & 1\leq k\leq n-1 \end{array}\right.$$

(7) $$C_{k}=\frac{C}{2^{n-k-1}},0\leq k\leq n-1$$

(3) The dynamic path loss probability calculation formula is as follows:

(8) $$\mathrm{current}\text{\_}\mathrm{prob}=\mathrm{initial}\text{\_}\mathrm{prob}+(\mathrm{final}\text{\_}\mathrm{prob}-\mathrm{initial}\text{\_}\mathrm{prob})\times \left(\frac{epoch}{\max\_epoch}\right)$$

### 2.5. SSAM Attention Mechanism

The improved SSAM has further lightweight design for CBAM [32,33]. Compared with CBAM’s original channel attention mechanism, SimAM [34] does not rely on global pooling and MLP but dynamically adjusts the weight of the feature map by calculating the statistical characteristics independent of the pixel distribution of each channel so that SSAM is more lightweight. At the same time, it can better capture the local features in the channel when the color of the water changes greatly (such as clear water, turbid water, or reflective water). Avoid the characteristics of small-scale floating objects on the water surface being diluted by global pooling to adapt to the detection of floating objects under different water colors.

**Figure 6:**
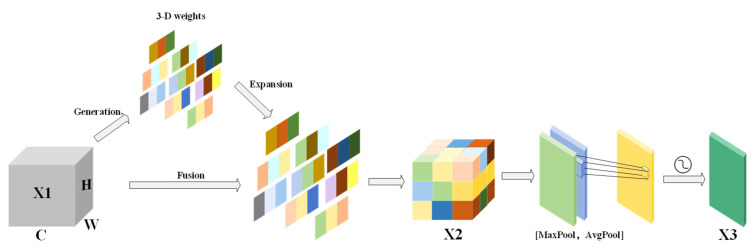
SSAM structure diagram.

The SSAM structure is shown in Figure 6. Initially, SimAM uses unbiased estimation to determine the pixel count in the feature map. The height and width of the feature map are represented by H and W, respectively. The formula is as follows:

(9) n=H⋅W−1

Then, the mean $\mu_{b,c}$ and variance ${\sigma^{2}}_{b,c}$ of each pixel of the input feature map X1 are calculated, and the three-dimensional attention weight y of the feature map is directly derived to generate a weight matrix. The attention weight is weighted with the original feature map X1, and the enhanced feature map X2 is obtained by pixel-by-pixel multiplication. The calculation formula is as follows:

(10) $$\mu_{b,c}=\frac{1}{H\cdot W}\sum_{h=1}^{H}\sum_{w=1}^{W}x_{b,c,h,w}$$

(11) $${\sigma^{2}}_{b,c}=\frac{\sum_{h=1}^{H}\sum_{w=1}^{W}{\left(x_{b,c,h,w}-\mu_{b,c}\right)}^{2}}{n}$$

(12) $$y=\frac{{\left(x-\mu \right)}^{2}}{4\cdot (\sigma^{2}+\lambda )}+0.5$$

(13) X2=X1⋅Sigmoid(y)

Among them, λ is a zero-proof positive number, and 0.5 is the offset, so that the value range of the attention weight is between (0, 1).

Finally, the spatial attention module takes the channel-enhanced feature map X2 and does average pooling and maximum pooling on it to make two single-channel feature maps. It then stitches these two maps together to focus on the global information and the local important float features. After a 7 × 7 convolution operation and Sigmoid activation function, while reducing the computational overhead, the key spatial information is retained, the spatial attention weight is generated, and a comprehensive spatial attention feature map X3 is output. The calculation formula of spatial attention is as follows:

(14) $$M_{s}(X2)=\sigma (f^{7\times 7}([AvgPool(X2);MaxPool(X2)]))$$

This combination mechanism of global and local feature enhancement can highlight the important characteristics of floating objects on the water surface, suppress background noise, improve the sensitivity of SSAM to small-scale floating objects under different water color backgrounds, and further help the model focus on the area where the floating objects are located, rather than being disturbed by the overall change of water color, which effectively improves the robustness of the model in complex scenes.

### 2.6. Small Target Detection Layer

**Figure 7:**
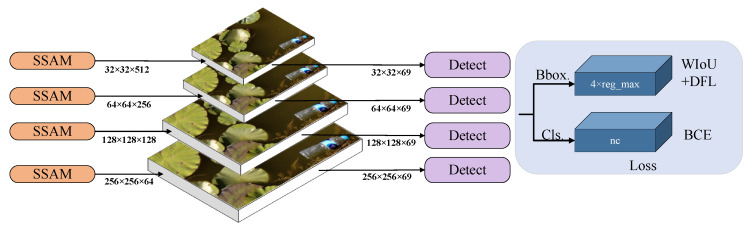
Small target detection layer structure diagram.

The YOLOv8 target detection layer enhanced the accuracy and classification recognition ability of the model for small target positioning by adaptively adjusting the size of the detection frame and suppressing background noise, so as to improve the accuracy of target detection. However, in aerial images, water surface floating objects usually appear in smaller proportions and variable shapes. Due to the large down-sampling multiple of YOLOv8 and the small size of the deep feature map, although its large receptive field can obtain more complex semantic information, there are still deficiencies in the expression of local details, which makes it difficult to meet the current detection requirements for very small surface floats. Therefore, this paper proposes the addition of a 256 × 256 small target detection layer to detect small floating objects below 4 × 4, with the aim of enhancing the model’s detection accuracy for floating objects of varying sizes in diverse scenarios. The structure of the small target detection layer is shown in Figure 7.

### 2.7. WIoU Loss Function

**Figure 8:**
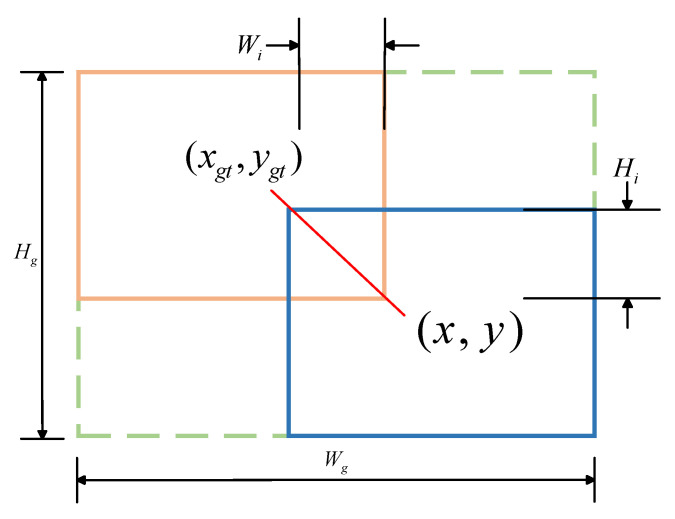
WIoU parameter diagram. Orange represents the ground truth bounding boxes, while blue represents the predicted bounding boxes.

The loss function optimizes the position error between the prediction box and the real box, generating an output prediction box that is close to the actual bounding box. In the training process of a neural network model, the loss function used has a significant impact on the training effect of the model. In this paper, WIoU with a dynamic non-monotone focusing mechanism is used as a new loss function. The WIoU parameter diagram is shown in Figure 8, where $H_{g}$ and $W_{g}$ are the length and width of the target detection box, $H_{i}$ and $W_{i}$ are the length and width of the intersection of IoU, and $\;(x_{gt},y_{gt})$ is the center point of the real box.

The core idea is to use “outlier” to evaluate the quality of the anchor frame. The smaller the outlier degree is, the higher the quality of the anchor frame is. Based on this, a reasonable gradient gain allocation strategy is designed. This strategy can reduce the competitiveness of high-quality anchor frames and reduce the harmful gradient generated by low-quality examples to adapt to the detection of floating objects with different shapes in complex environments. The formula is as follows:

(15) $$L_{WIoU}=r\cdot \exp[\frac{{\left(x-x_{gt}\right)}^{2}+{\left(y-y_{gt}\right)}^{2}}{{\left(W_{g}^{2}+H_{g}^{2}\right)}^{*}}]\cdot (1-\mathrm{IoU})$$

(16) $$r=\frac{\beta }{\delta \cdot \alpha^{\beta -\delta }}$$

(17) $$\beta =\frac{{\left(1-\mathrm{IoU}\right)}^{*}}{1-\bar{\mathrm{IoU}}}\in [0,+\infty )$$

Here, r denotes the gradient gain, β denotes the outlier degree, and α and δ are hyperparameters; when β = δ and r = 1, the anchor frame will achieve the highest gradient gain, where $1-\bar{\mathrm{IoU}}$ is the moving average of m.

## 3. Results

### 3.1. Model Evaluation Indicators

To precisely assess the model’s performance, the experiments employed Average Precision (*AP*) for each category, mean Average Precision (*mAP*) across all categories with Intersection over Union (IoU) thresholds of 0.5 and 50–95, miss rate, F1 score, Frames Per Second (FPS), and Giga Floating Point Operations Per Second (GFLOPs) as metrics indicative of the model’s efficacy. The following formulas were used for computation:

(18) $$AP=\int_{0}^{1}P(R)dR$$

(19) $$mAP=\frac{\sum_{i=0}^{n}AP(i)}{n}$$

(20) $$\mathrm{missrate}=\frac{FN}{TP+FN}\approx 1-\mathrm{recall}$$

(21) $$F1=2\times \frac{\mathrm{Precision}\times \mathrm{Recall}}{\mathrm{Precision}+\mathrm{Recall}}$$

In the formula, A signifies the area enclosed by the *P*–*R* curve and the coordinate axes; B indicates the number of categories; C denotes the quantity of samples accurately classified as positive class targets; D is the number of samples in which positive class targets are erroneously classified as negative.

### 3.2. Ablation Experiment

In order to verify the effectiveness of each improved method proposed in this paper, the detection effects of different improved methods are evaluated by ablation experiments based on YOLOv8n, as shown in Table 1.

The experimental data in Table 1 clearly show that each improved method’s performance expression is significantly better than that of the benchmark model. Group A is the original YOLOv8n model. In group B, the HorBlock_G module replaced the C2f module of layers 4 and 8 in the backbone network of group A, increasing the mAP50 and mAP50-95 by 4.9% and 8.8%, respectively, and reducing the miss rate by 3%. This effectively verified the model’s performance in extracting finer-grained floating object features. After replacing the C2 f module in the second and sixth layers of the backbone network of group B with the C3 module in group C, the mAP50 is only 0.4% higher than that of group B. However, its GFL0Ps and FPS/s values decrease and increase by 0.3 G and 5.8 s, respectively, which is in line with the improvement idea of reducing the amount of backbone network computing to use deeper network design. In group D, three SSAM attention mechanisms are introduced in the neck part, which achieves effective performance improvement while lightweighting the algorithm. The algorithm increases the mAP50 and mAP50-95 by 3.6% and 5.4%, respectively, while reducing the miss rate by 2%.

On the basis of the improved methods of group C and group D, group E adds a small target detection layer so that the model can more accurately identify the small floating objects below 4 × 4 in the water surface image. This improvement introduces a certain amount of computational overhead to the model. But the improved method increases the model’s mAP50 and mAP50-95 values by 11.4% and 11.6%, respectively, compared to the benchmark model, and it reduces the miss rate by 12%. This significantly enhanced the model’s detection and classification performance for floating objects on the water surface, thereby meeting the objective of improving the detection accuracy of floating objects. Finally, group F replaced the CIoU of group E with the WIoU loss function. While maintaining the calculation amount no longer increasing, the mAP50, mAP50-95, and FPS values were slightly improved compared with group E. The experimental results show that all the improved methods have effectively improved the detection accuracy of floating objects on the water surface.

### 3.3. Loss Function Comparison Experiment

**Figure 9:**
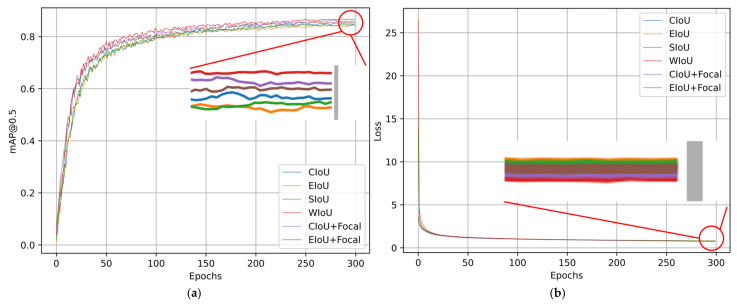
Comparison of mAP50 and loss convergence of different loss functions diagram. (**a**) The mAP50 of different loss functions; (**b**) loss convergence of different loss functions.

In order to evaluate the influence of different loss functions on the performance of the model, based on the YOLOv8-HSH algorithm, the mAP50 and loss convergence of WIoU and different loss functions (including CIoU, EIoU, SIoU, CIoU + Focal, and EIoU + Focal) are compared, as shown in Figure 9.

The three loss functions exhibiting superior performance in Figure 9a are intuitively attributable to their dynamic adjustment mechanisms. Nevertheless, the gradient gain allocation method of the WIoU loss function proves to be more efficacious. This is further corroborated by Figure 9b. Despite WIoU not exhibiting the most rapid reduction in loss gradient, it consistently refines the assessment approach for candidate box quality during subsequent training, ultimately minimizing the loss value to an optimal level, hence demonstrating the efficacy of this algorithmic enhancement.

### 3.4. Contrast Experiment

To further validate the efficacy of the improved YOLOv8-HSH algorithm, under identical parameter indices, a comparison was conducted with algorithms such as YOLOv5-SF [22], YOLOv5-SC [16], YOLOv5s-CDF [27], YOLOv5-ECB [26], YOLOv8n, CDW-YOLOv8 [17], YOLOv10l, among others. The experimental results are shown in Table 2.

Data from Table 2 indicate that the YOLOv8-HSH algorithm enhanced mAP50 and mAP50-95 by 11.7% and 12.4%, respectively, relative to the original model YOLOv8n. The miss rate diminishes by 11%, the F1 score ascends by 11%, and the AP value for each category rises by a minimum of 5.6%. In comparison to YOLOv5l, YOLOv5-SF [22], YOLOv8l, and YOLOv10l algorithms, there is a significant reduction in the miss rate, alongside an increase of over 2.1% in the mAP50, AP value, and F1 score for each category. The YOLOv8-HSH method is compared with the YOLOv5-SC [16] and YOLOv5-ECB [26] algorithms, both of which incorporate a minor target detection layer. Despite the proximity of the mAP50 accuracy values and F1 scores across the three models, the YOLOv8-HSH algorithm exhibits a smaller size and superior detection time. The detection accuracy of floating objects on the water surface is markedly improved under tougher thresholds. The mAP50-95 value exceeds the previous one by 2.6%, surpassing the YOLOv5-SC [16] and YOLOv5-ECB [26] algorithms. In comparison to the YOLOv8-HSH method, the CDW-YOLOv8 [17] algorithm has a superior detection speed and a smaller model. However, its detection accuracy significantly lags behind that of the YOLOv8-HSH algorithm, rendering it inadequate for the task of detecting floating objects on water surfaces. In comparison to the YOLOv5s-CDF [27] algorithm, which is likewise utilized for aerial picture recognition, the YOLOv8-HSH algorithm exhibits a more compact model, improved detection velocity, and enhanced performance metrics. Relative to the YOLOv5s-CDF [27] method, the YOLOv8-HSH algorithm demonstrates a 2.4% enhancement in mAP50, a 4% improvement in mAP50-95, a 2% reduction in the miss rate, and a 2% rise in the F1 score. Thus, it is better suited for identifying floating objects in intricate situations. In conclusion, compared to other floating object detection algorithms currently in use, the YOLOv8-HSH algorithm performs better when it comes to aerial images of floating objects in complex environments.

### 3.5. Validation Experiment

To evaluate the effectiveness of the improved algorithm, we conducted comparative tests with other algorithms on the publicly available IWHR_AI_Label_Floater_V1 [35], VisDrone2019 [36], and SeaDroneSee [37] datasets. The input image size for all experiments was set to 640 × 640.

**Figure 10:**
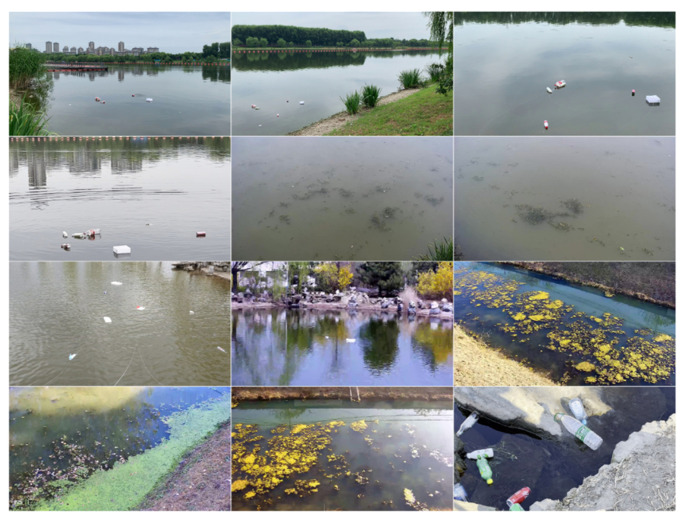
The sample images of the IWHR_AI_Label_Floater_V1 dataset.

The sample images of the IWHR_AI_Label_Floater_V1 dataset are shown in Figure 10. The IWHR_AI_Label_Floater_V1 [35] dataset was created by the China Institute of Water Resources and Hydropower Research to further the utilization of artificial intelligence technologies within China’s water resources sector. The collection comprises 3000 open-source photographs of floating objects, photographed from many angles, in varying lighting situations, and set against a range of backgrounds. Mobile phones, digital cameras, and surveillance cameras with different resolutions were used to take these pictures. The dataset is not only abundant in samples but also extremely authoritative. This article adheres to the dataset division ratio specified by the author and employs a random batch processing technique for dataset division. The experimental results are shown in Table 3.

The comparative experiments conducted on the publicly accessible IWHR_AI_Label_Floater_V1 dataset demonstrate that the YOLOv8-HSH algorithm enhanced mAP50 and mAP50-95 by 2.4% and 2.6%, respectively, relative to the baseline model YOLOv8n, while the miss rate diminished by 3%, indicating a substantial improvement.

**Figure 11:**
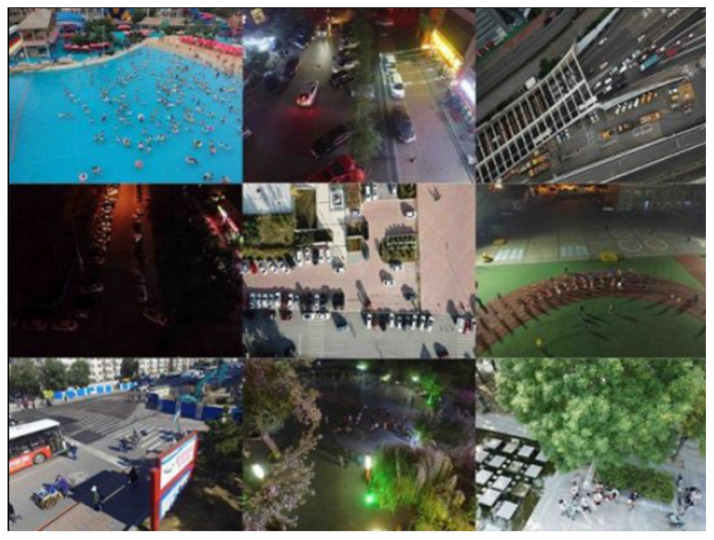
The sample images of the VisDrone2019 dataset.

While the aforementioned comparative experiments have effectively demonstrated the efficacy of the improved algorithm presented in this research, IWHR_AI_Lable_Floater_V1 is not an aerial dataset. The height and angle of the shot significantly differ from the dataset presented in this research, and the detection category is rather limited. To further validate the efficacy of the algorithm presented in this research, this paper compares the YOLOv8-HSH algorithm with the YOLOv8n and YOLOv5-CN [15] algorithms on the VisDrone2019 [36] public aerial dataset. The sample images of the VisDrone2019 dataset are shown in Figure 11. VisDrone2019 was captured by a research team from Tianjin University using different models of drones in 14 different cities and rural areas in China, under various background scenes, weather conditions, and lighting conditions. The dataset is separated into ten label groups, with road traffic serving as the primary shooting object. It is frequently employed for the identification of aerial tiny targets and the validation of classification and recognition algorithms, possessing considerable authority. The experimental results are shown in Table 4.

From the experimental results in Table 3 and Table 4, it can be observed that the YOLOv8-HSH algorithm demonstrates a greater performance advantage over the baseline model YOLOv8n when processing aerial datasets. Compared to YOLOv8n, YOLOv8-HSH achieves a 4.4% improvement in mAP50, along with significant increases in detection accuracy across all categories. Additionally, when compared to the YOLOv5-CN [15] algorithm, which also incorporates an additional small object detection layer, YOLOv8-HSH not only improves mAP50 by 3.5% but also reduces model complexity by 2.4 G. The experimental results indicate that YOLOv8-HSH performs exceptionally well in addressing the challenges posed by aerial images, where small objects constitute a minimal proportion and key features are severely weakened. This advantage is not only attributed to the introduction of the small object detection layer but is also closely related to the application of HorBlock_G.

**Figure 12:**
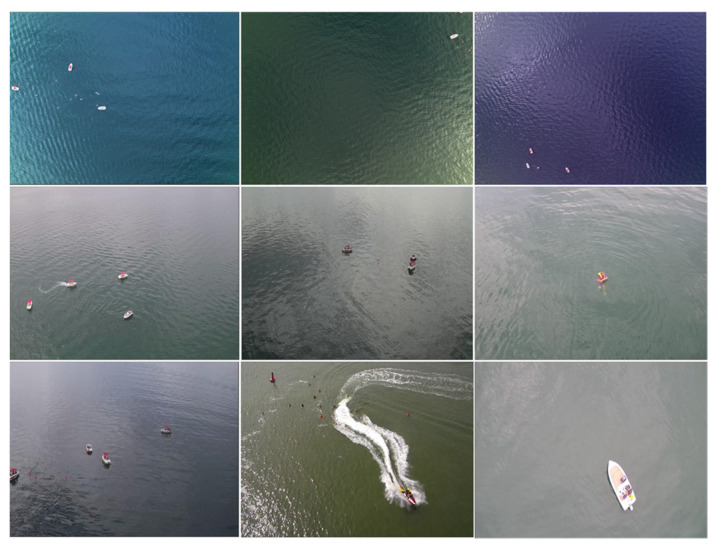
The sample images of the SeaDroneSee dataset.

To further validate the effectiveness of the improved algorithm proposed in this paper and its application value in fields such as environmental monitoring and maritime rescue, we conducted comparative experiments with other algorithms on the public SeaDronesSee dataset. The SeaDronesSee dataset was released by Varga et al. from the University of Tübingen, Germany, at the WACV conference in 2022, aiming to advance the use of drones in search and rescue operations at sea. The dataset includes 8930 training images, 1547 validation images, and 3750 test images, covering various lighting conditions, shooting distances, and angles. It fully considers factors such as small target sizes and complex environments. Example images from the SeaDronesSee dataset are shown in Figure 12.

The experimental results show that, thanks to the application of the SSAM model, the improved YOLOv8-HSH algorithm exhibits greater advantages on the aerial dataset with diverse water body colors compared to the experiments conducted on the aforementioned public datasets. As shown in Table 5, YOLOv8-HSH achieves a 10.6% improvement in mAP50 over the baseline model YOLOv8n, particularly excelling in detecting small objects (such as Life_saving_appliances and buoy) that YOLOv8n struggles with. Furthermore, compared to the DFLM-YOLO algorithm, which is specifically designed for the SeaDroneSee dataset, the YOLOv8-HSH algorithm also demonstrates notable performance advantages. Although the DFLM-YOLO algorithm achieves higher detection accuracy for larger targets (such as Swimmer, Boat, and Jetski) than the YOLOv8-HSH algorithm, the YOLOv8-HSH algorithm significantly outperforms DFLM-YOLO in detecting extremely small targets (such as life-saving equipment and buoys), while its computational complexity is considerably lower than that of DFLM-YOLO. From the perspective of practical applications, lower algorithm complexity and high detection accuracy for extremely small targets not only help increase the flight altitude of drones, expand the detection range, and improve inspection efficiency but also enable a more timely detection of people in distress during maritime inspections.

### 3.6. Visual Analysis of Test Results

**Figure 13:**
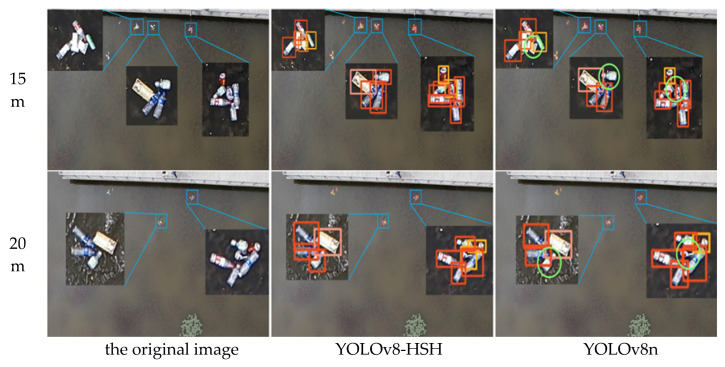
Scene map with dense floating objects. The bright green circles indicate missed detections.

**Figure 14:**
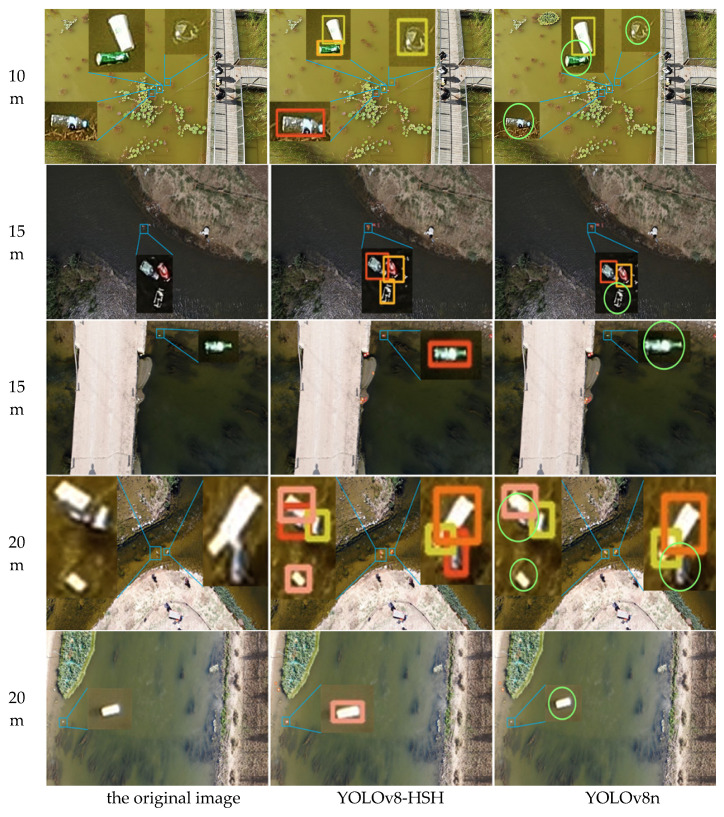
Scene maps of floating objects obtained under different aerial photography heights and water color conditions. The bright green circles indicate missed detections.

**Figure 15:**
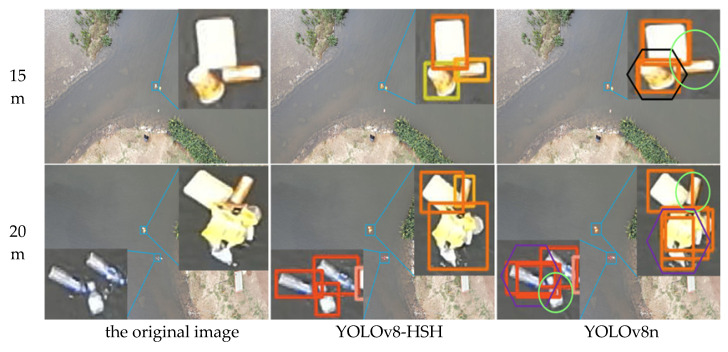
Scene maps where multiple floating objects are difficult to identify due to similar adjacent colors. The black hexagonal boxes indicate false detections, the purple hexagonal boxes represent redundant detection boxes, and the bright green circles indicate missed detections.

In order to more intuitively reflect the detection and classification efficacy of the YOLOv8-HSH algorithm, both the YOLOv8-HSH and the original YOLOv8n model are employed to visualize detection outcomes across varying aerial photography altitudes and circumstances, as depicted in Figure 13, Figure 14 and Figure 15. Among them, the bright green circle indicates that the target is missed, the purple hexagon frame indicates that the detection frame is redundant, the black hexagon frame indicates the wrong detection, the red rectangle label indicates the bottle, the meat pink rectangle label indicates the carton, the orange red indicates the plastic bag, the orange yellow rectangle label indicates the drink can, the yellow green rectangle label indicates the plastic cup, and the denser label is locally enlarged. Comparative analysis reveals that YOLOv8n exhibits significant missed and erroneous detections, whereas the improved YOLOv8-HSH algorithm demonstrates adaptability to complex environments at varying altitudes, yielding superior detection performance in scenarios involving dense small targets and fluctuating water color.

The scene of dense distribution of floating objects is a classic problem in the field of detection, classification, and recognition of floating objects on the water surface. As shown in Figure 13, profit from the application of the HorBlack_G module, the improved YOLOv8-HSH model has greatly improved the detection ability of dense floating objects compared with the YOLOv8n model. It effectively reduces the miss rate of the model by means of multi-gradient and multi-scale feature superposition enhancement.

Figure 14 shows different water color scenes at different heights. Adapting to the detection of color floating objects in multiple waters at different heights is an important manifestation of the model in line with practical applications. Compared with the YOLOv8n model, the YOLOv8-HSH algorithm effectively enhanced the detection performance of the model for small floating objects by adding a small target detection layer while using SSAM to suppress background noise. The phenomenon of missed detection is greatly reduced, which significantly improves the detection and classification ability of the model for color scenes at different heights and different waters.

Figure 15 is a multi-adjacent color floating object scene. In the scene of multi-adjacent color floating objects, detection frame redundancy and false detection and missed detection often occur. The plastic cup with a yellow–green label in the image was repeatedly misidentified as a can with a light-orange label. It can be seen that the detection and classification ability of the model is seriously disturbed by the multi-adjacent color floating objects. Compared with the YOLOv8n model, the YOLOv8-HSH algorithm optimizes the traditional IoU loss function through the gradient gain allocation strategy, which effectively enhanced the model’s ability to locate and classify floating objects so that it shows the best performance in the above model.

It can be seen that the YOLOv8-HSH algorithm improves the feature extraction ability and target positioning ability of the model by HorBlack_G, SSAM, and WIoU and adding a small target detection layer. It effectively reduces the missed detection rate and false detection rate of the model, realizes the accurate detection and classification recognition of floating objects, and confirms the effectiveness of the improved algorithm in this paper.

## 4. Conclusions and Prospects

Timely detection and the removal of floating debris in rivers and lakes is not only an important measure to protect water resources in lakes and rivers but also an effective way to reduce the pressure of marine litter management at its source. However, traditional methods such as manual detection and monitoring with surveillance cameras are inefficient, costly to maintain, and fail to meet the practical needs of river inspections. Therefore, this paper leverages the efficiency and convenience of UAV-based inspections and proposes an improved algorithm, YOLOv8-HSH, to detect and classify floating debris on water surfaces from an aerial perspective. The algorithm is designed to address the challenges of UAVs autonomously navigating riverbanks with varying heights and detecting floating debris under diverse backgrounds, weather conditions, and lighting scenarios.

In this study, we simulated real-world water surface environments and constructed an aerial-based floating debris dataset, with UAV flight altitudes set at 10 m, 15 m, and 20 m. Considering the challenges posed by the small size of floating debris in aerial images, the weakened saliency of their features, and the impact of lighting variations and dense distributions of debris on detection accuracy, we optimized the network structure and loss function of the YOLOv8n algorithm. These improvements enabled the YOLOv8-HSH algorithm to achieve a mean Average Precision (mAP) of 90% on the self-constructed floating debris dataset. Compared to the baseline model, the YOLOv8-HSH algorithm achieved an 11.7% improvement in mAP50 and an 11% reduction in the missed detection rate. In complex water surface environments, the algorithm demonstrated superior detection and classification performance, as well as strong robustness and generalizability. Improving the model’s capability to detect extremely small targets and its ability to identify small objects against varying water color backgrounds not only helps increase the flight altitude of drones, expand the detection range, and enhance detection efficiency but also improves the model’s adaptability to diverse water environments. This advancement is of significant importance for floating object detection on water surfaces and offers substantial application value in areas such as maritime rescue and environmental monitoring. However, we also observed that the model still struggles with challenging samples, highlighting the need to further expand the scale and diversity of the dataset, which will be a key focus of future research.

In future studies, we aim to continuously enrich the dataset, focusing on images with motion blur caused by UAV vibrations and samples where strong reflections result in severe feature loss and the merging of floating debris. We will conduct more detection and classification experiments and further improve the algorithm to reduce the false-positive and false-negative rates for difficult-to-identify samples while maintaining current performance levels. These efforts aim to contribute to the intelligent implementation of the “River Chief System”.

## Figures and Tables

**Table 1:** Ablation experimental results. The bolded portions indicate the optimal results in each column of metrics.

Group	YOLOv8n	HorBlock_G	C3	SSAM	Detect Layer	WIoU	mAP50 /%	mAP50-95 /%	F1 Score	Miss Rate/%	FPS/s	GFL0Ps
A	√						78.3	52.7	0.81	17	270	**8.1**
B	√	√					83.2	61.5	0.83	14	238.1	9.4
C	√	√	√				83.6	62.2	0.84	14	243.9	9.1
D	√			√			81.9	58.1	0.81	15	277.8	8.4
E	√	√	√	√	√		89.7	64.3	0.87	5	238.1	13.5
F	√	√	√	√	√	√	**90**	**6** **5.1**	**0.87**	**5**	**245.82**	13.5

**Table 2:** Comparison of experimental results with other algorithms on the self-built dataset in this paper. The bolded portions indicate the optimal results in each column of metrics.

Model	mAP50 /%	mAP50-95/%	AP/%					Miss Rate/%	F1 Score	FPS/s	GFL0Ps
			Bott-Le	Carton	Plast-Ic Bag	Drink Can	Plast-Ic Cup				
YOLOv5l	83.3	61.6	91.1	84.8	85.4	69.2	86	12	0.83	132.33	107.7
YOLOv5-SF [22]	80.6	56.7	87.3	82.5	82.5	66.5	84	14	0.80	235.1	15.9
YOLOv5-SC [16]	88.9	62.5	93.8	92.2	86.6	82.1	89.7	5	0.86	192.12	19.3
YOLOv5s-CDF [27]	87.6	61.1	94.1	91.2	85.3	77.5	89.8	7	0.85	173.5	21.3
YOLOv5-ECB [26]	88.8	62.1	94.6	93.1	86.4	79.8	90.1	5	0.86	197.2	18.6
CDW-YOLOv8 [17]	83	55.7	91.3	86.9	85	65.5	85.9	10	80	**297.62**	10.1
YOLOv8n	78.3	52.7	84.8	83.7	82.5	59.9	80.6	16	0.76	270	**8.1**
YOLOv8l	84.3	65.1	90.4	87	85.8	70.3	88	15	0.82	114.38	165.3
YOLOv10l	84.7	63.8	90	87.4	84.9	74.2	86.8	11	0.84	143.88	126.4
YOLOv8-HSH	**90**	**6** **5.1**	**94.7**	**93.7**	**88.1**	**82.8**	**90.1**	**5**	**0.87**	245.82	13.5

**Table 3:** Comparative experiments on public dataset IWHR_AI_Label_Floater_V1. The bolded portions indicate the optimal results in each column of metrics.

Model	mAP50/%	mAP50-95/%	Miss Rate/%	GFL0Ps
YOLOv8n	89.2	64.7	19	**8.1**
YOLOv8-HSH	**91.6**	**67.3**	**16**	13.5

**Table 4:** Comparison of experimental results with other algorithms on public dataset VisDrone2019. The bolded portions indicate the optimal results in each column of metrics.

Model	mAP50 /%	AP/%										GFL0Ps
		Pedestrian	People	Bicycle	Car	Van	Truck	Tricycle	Awning-Tricycle	Bus	Mot-Or	
YOLOv5-CN [15]	35.1	41.1	32.8	11.3	74	35.9	31.2	24	12.6	48.3	40.3	15.9
YOLOv8n	34.2	35.9	28.8	8.5	76.1	39.6	32.3	24.1	12.1	46.1	38.2	**8.1**
YOLOv8-HSH	**38.6**	**44.3**	**38.2**	**11.9**	**81**	**43.9**	**32.5**	**24.7**	**12.9**	**52.3**	**44.3**	13.5

**Table 5:** Comparison of experimental results with other algorithms on public dataset SeaDroneSee-V2. The bolded portions indicate the optimal results in each column of metrics.

Model	mAP50 /%	AP/%					GFL0Ps
		Swimmer	Boat	Jetski	Life_Saving_Appliances	Buoy	
DFLM-YOLO [38]	76.6	**78.3**	**95.5**	**86.3**	45.8	77.3	23.3
YOLOv8n	66.2	69.8	91	83.8	28.6	57.8	**8.1**
YOLOv8-HSH	**76.8**	77.4	95.3	80.3	**50.3**	**80.7**	13.5

## Data Availability

All data used in this paper can be obtained by contacting the authors of this study.
